# pH-Tolerant Wet Adhesion of Catechol Analogs

**DOI:** 10.1021/acsami.4c01740

**Published:** 2024-04-15

**Authors:** George D. Degen, Syeda Tajin Ahmed, Parker R. Stow, Alison Butler, Roberto C. Andresen Eguiluz

**Affiliations:** † Department of Chemical Engineering, University of California, Santa Barbara, California 93106, United States; ‡ Department of Materials Science and Engineering, University of California, Merced, California 95344, United States; § Department of Chemistry and Biochemistry, University of California, Santa Barbara, California 93106, United States; ∥ Health Sciences Research Institute, University of California, Merced, California 95344, United States

**Keywords:** wet adhesion, DOPA, HOPO, pH tolerant adhesive, mussel-inspired, surface forces apparatus, surface primers

## Abstract

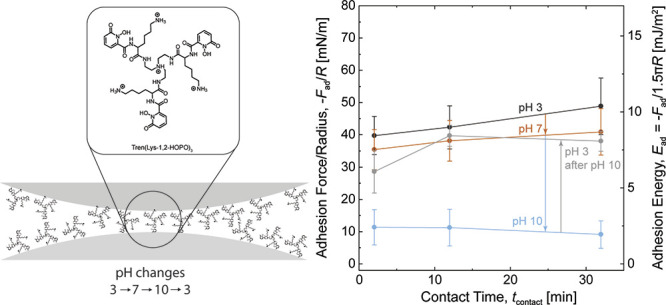

The need for improved wet adhesives has driven research on mussel-inspired materials incorporating dihydroxyphenylalanine (DOPA) and related analogs of the parent catechol, but their susceptibility to oxidation limits practical application of these functionalities. Here, we investigate the molecular-level adhesion of the catechol analogs dihydroxybenzamide (DHB) and hydroxypyridinone (HOPO) as a function of pH. We find that the molecular structure of the catechol analogs influences their susceptibility to oxidation in alkaline conditions, with HOPO emerging as a particularly promising candidate for pH-tolerant adhesives for diverse environmental conditions.

## Introduction

Biomedical and marine adhesives, sealants, and coatings must bind to surfaces in the presence of water, a challenge that thwarts conventional adhesives.^1^ To overcome this challenge, researchers have taken inspiration from the adhesives produced by marine organisms including mussels^2^ and sandcastle worms,^3^ which are rich in the amino acid 3,4-dihydroxyphenylalanine (DOPA). DOPA and related analogs of the parent catechol form adhesive^4^ and cohesive^5^ interactions and have been explored for use in diverse applications.^1,6,7^ However, a major limitation of catechol-based materials is the tendency of many catechol analogs to oxidize in neutral to alkaline conditions, including physiological and marine environments. Catechol analog oxidation enables covalent bonding but weakens reversible interactions^8,9^ and complicates the storage, delivery, and longevity of catechol-based materials. Control of catechol analog oxidation is therefore needed to expand the scope of applications of catechol-based materials.

The susceptibility of catechol analogs to oxidation is influenced by their molecular structure. For example, sandcastle worm cement contains catechols substituted with chlorine,^10^ and many siderophores contain dihydroxybenzamide (DHB)^11,12^ and quinoline derivatives,^13^ all of which resist oxidation in neutral to mildly alkaline conditions. Oxidation-resistant catechol analogs have also been used in synthetic materials including gels,^14−17^ coatings,^18−22^ and particle stabilizers.^23−25^ Of the catechol analogs, hydroxypyridinone (HOPO) is noteworthy for its ability to form tris coordination complexes with metal ions at neutral pH, which can drive gelation in physiological environments,^26,27^ whereas other catechol analogs require alkaline conditions for gelation.^28^ DHB, and particularly 3,4-DHB, is also noteworthy for its structural similarity to DOPA but superior resistance to oxidation.^8^ These properties make HOPO and DHB promising candidates for pH-tolerant materials.

In contrast with the established understanding of the adhesion of oxidation-prone catechol analogs,^4^ the adhesion of oxidation-resistant catechol analogs is poorly understood. Although macroscopic adhesion of materials functionalized with HOPO^16,29^ and DHB^30−34^ has been reported, molecular level adhesion of HOPO has not been investigated, and questions remain about effect of environmental conditions on the adhesion of DHB. We previously found lower adhesion of compounds functionalized with 2,3-DHB in neutral conditions than in acidic conditions,^32^ but did not investigate the origin of the pH-dependence. Furthermore, the 2,3-DHB and 3,4-DHB functionalities show different susceptibilities to oxidation in solution,^8^ but their environmentally dependent adhesion has not been compared. Rational design of catechol-based adhesives requires a better understanding of the effects of molecular structure and environmental conditions on the adhesion of catechol analogs.

Here, we report the adhesion of surface primers functionalized with the catechol analogs 1,2-HOPO, 3,4-DHB, and 2,3-DHB, as a function of pH. Nanoscale films of the surface primers were deposited on mica surfaces, and the adhesion between the surfaces was measured using a surface forces apparatus (SFA). We find that the surface primers adhere to mica surfaces in acidic conditions and that adhesion decreases upon exposure to alkaline conditions. Returning to acidic conditions restores the adhesion of the primers to different extents, which we explain by considering both deprotonation and catechol analog oxidation. Our results give insight into the adhesion and oxidation of catechol analogs toward the development of pH-tolerant wet adhesives for use in diverse environments.

## Experimental Section

Details on synthesis and characterization of Tren(Lys-1,2-HOPO)_3_ and Tren(Lys-3,4-DHB)_3_ are included in the Supporting Information S1, Figures S1–S6, and Tables S1 and S2. Tren(Lys-2,3-DHB)_3_ was synthesized according to published protocols.^32^ Each compound was stored at 1 mM in an acetate solution (50 mM acetate, 150 mM KNO_3_, pH 3) at 4 °C before use to avoid oxidation. Surface primer films were deposited on the mica surfaces following a method developed previously.^32−34^ Mica surfaces in the SFA were incubated in a meniscus of an acidic primer solution (90 μM primers, 50 mM acetate, 150 mM KNO_3_, pH 3) for at least 30 min. To change the pH of the solution, the surfaces were removed from the SFA, rinsed with several mL of buffer at pH 7 (50 mM phosphate, 150 mM KNO_3_) or pH 10 (50 mM carbonate, 150 mM KNO_3_), and remounted in the SFA, a process that introduces an uncertainty of approximately 1 nm to the distance measurement.^35^ A water bath was placed at the bottom of the SFA chamber to maintain a saturated vapor environment and limit evaporation of the meniscus between the surfaces during force measurements.

Force measurements of films of surface primers were performed with a surface forces apparatus (SFA2000, SurForce LLC), the full details of which are described elsewhere.^36^ Cleaved mica surfaces (thickness 3–12 μm) were backsilvered, glued (EPON 1004F, Miller-Stephenson) onto cylindrical glass disks (radius of curvature *R* = 2 cm), and arranged in the SFA in a crossed cylinder geometry, with one surface suspended on a double cantilever spring (spring constant ∼1000 N/m). White light multiple beam interferometry was used to measure the distance between the mica surfaces, with zero separation distance determined by contact between the mica surfaces in air. The interference fringes were used to infer the deflection of the spring and therefore the normal force *F*. In all plots, the force is normalized by the average radius of curvature *R* of the surfaces at the contact point, measured by the interference fringes. By convention, positive forces indicate repulsion between the surfaces, and negative forces correspond to attraction. The surfaces were brought into contact, compressed, and separated by translating the base of the cantilever spring at a constant speed (15–25 nm/s). The surfaces were held at maximum compression for a dwell time of 0, 10, or 30 min. The total contact time *t*_contact_ was the sum of the time required to compress and separate the surfaces (∼2 min) and the dwell time. The maximum attractive force measured during separation was denoted as the adhesion force, −*F*_ad_/*R*. Adhesion energies *E*_ad_ were calculated using the Johnson–Kendall–Roberts (JKR) theory,^37^*E*_ad_ = −*F*_ad_/1.5π*R*, an analysis that assumes that the only influence of the molecularly thin adsorbed layer on the contact mechanics is to modify the surface energy. Deviation from JKR theory due to variations in glue and mica thicknesses in our layered surfaces^38^ is treated as a source of error. Error bars indicate the standard deviation of the measurements performed at three or more contact points. Total numbers of experiments with independent sets of mica surfaces are shown in Tables S3–S5 in the Supporting Information.

## Results and Discussion

**Figure 1:**
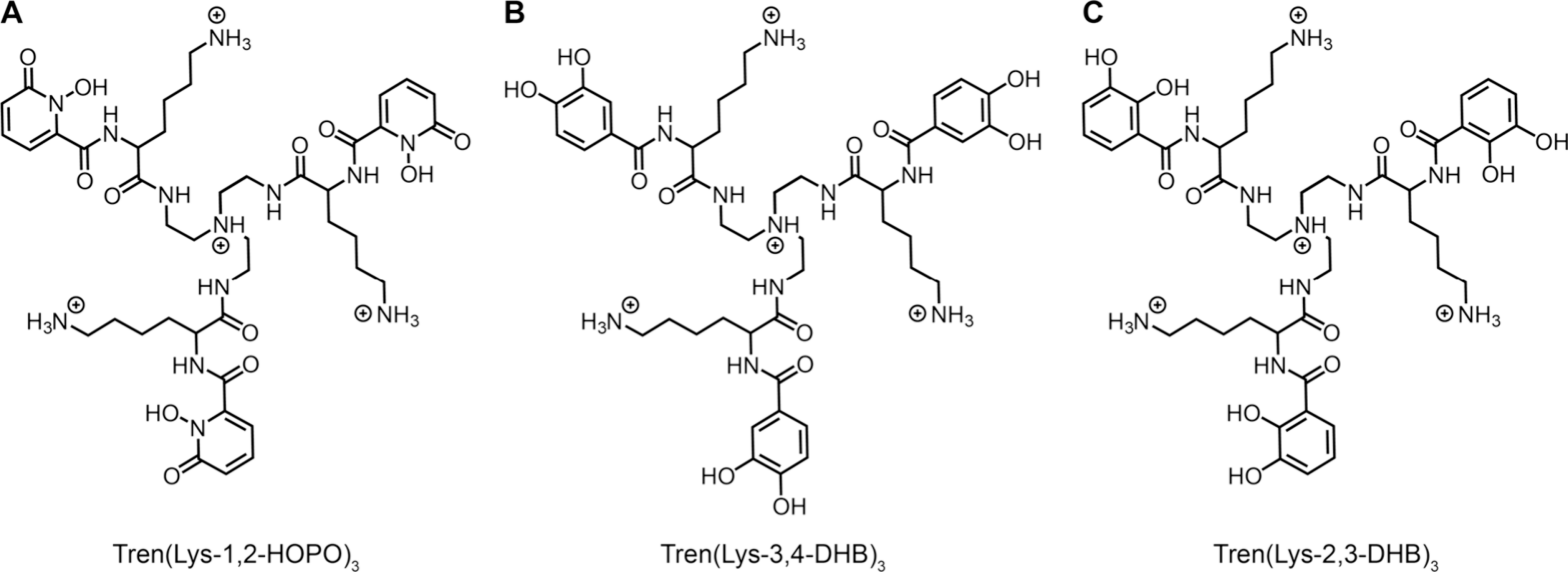
Surface primers functionalized with lysine and catechol analogs. (A) Tren(Lys-1,2-HOPO)_3_, (B) Tren(Lys-3,4-DHB)_3_, and (C) Tren(Lys-2,3-DHB)_3_.

We hypothesized that tuning the molecular structure of catechol analogs would enable robust, oxidation-resistant adhesion over a range of pH values. To test this hypothesis, we synthesized surface primers based on a tris(2-aminoethyl)amine (Tren) scaffold, a design strategy that was previously used to study molecular-level adhesion of catecholic surface primers.^32−34^ The study of surface primers, small adhesive molecules that bind to surfaces but lack mechanisms for energy dissipation, allows adhesive functionalities to be systematically varied while avoiding conformational complexity that complicates the interpretation of nanoscale adhesion measurements. The Tren scaffold was functionalized with catechol analogs, either 1,2-HOPO, 3,4-DHB, or 2,3-DHB. Lysine (Lys) was included to avoid aggregation and facilitate adsorption and adhesion on mica. We name the surface primers Tren(Lys-1,2-HOPO)_3_, Tren(Lys-3,4-DHB)_3_, and Tren(Lys-2,3-DHB)_3_, shown in Figure 1. The surface primers were deposited onto the mica surfaces by adsorption. Adhesion between the surfaces was measured with an SFA, a technique that allows direct and simultaneous measurement of the normal force and nanoscale separation distance between mica surfaces coated in molecularly thin films, thereby enabling molecular-level interpretation of experiments.^36^

**Figure 2:**
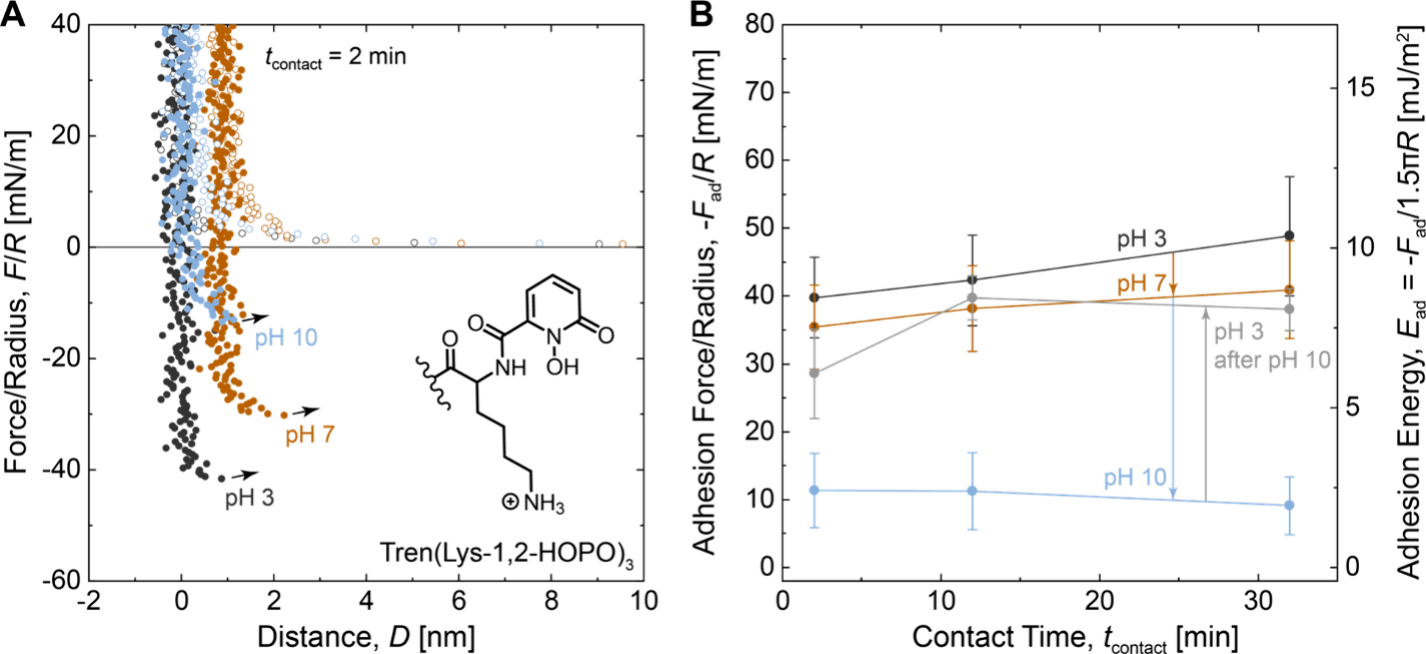
Effect of the pH on the adhesion of Tren(Lys-1,2-HOPO)_3_. (A) Plots of force/radius *F*/*R* vs mica–mica separation distance *D*. Open circles correspond to approach; closed circles correspond to separation. (B) Plots of adhesion force/radius −*F*_ad_/*R* and adhesion energy *E*_ad_ vs contact time *t*_contact_. Measurements were performed in solutions of 50 mM acetate (pH 3), 50 mM phosphate (pH 7), or 50 mM carbonate (pH 10), each with 150 mM KNO_3_.

We first investigated the adhesion of Tren-based surface primers functionalized with 1,2-HOPO, a functionality with desirable oxidation resistance and metal coordination properties,^26^ but not previously investigated for molecular level adhesion. Figure 2A shows a plot of force/radius vs mica–mica separation distance for films of Tren(Lys-1,2-HOPO)_3_ deposited at pH 3 (black circles). As the surfaces were brought into contact (open circles), repulsive forces were measured at mica–mica separation distances below 5 nm. As the compression increased, the film thickness reached a constant value below 1 nm. Upon separation of the surfaces (closed circles), the force became increasingly negative before abruptly dropping to zero with a corresponding abrupt increase in the mica–mica separation distance. The jump from contact indicates that the surface primers mediated adhesion between the mica surfaces. Interestingly, the value of the adhesion force was similar to the values previously measured for surface primers functionalized with 2,3-DHB and lysine,^32−34^ despite the different catechol analog. To test the influence of pH on adhesion, we exchanged the buffer solution between the primer-coated surfaces and repeated the force measurements, shown in Figure 2A for pH 7 (orange circles) and pH 10 (blue circles). Figure 2B shows the average adhesion force measured at each pH as a function of the contact time. The plots show that increasing the pH from 3 to 7 slightly decreased the adhesion force at each contact time. Further increasing the pH from 7 to 10 resulted in a dramatic drop in adhesion. Upon returning the pH from 10 to 3, the adhesion was substantially restored (Figure 2B, gray circles). Slight increases in adhesion force with contact time were observed at pH 3 and 7, likely due to the progressive formation of adhesive interactions. We note that Figure 2B combines data from pH sweep experiments (3 to 7 to 10) and reversibility experiments (3 to 10 to 3). Data from the reversibility experiments alone are included in Figure S7 and show complete recovery of the adhesion force upon return to pH 3 within experimental error. We also note that our experimental configuration yielded a contact radius of order 10 μm.^39^ Consequently, the reported adhesion energies correspond to an average over many nanoscale interactions. These adhesion energies are much lower than the values typically reported for commercial adhesives, but the discrepancy is expected based on the inability of the surface primers to dissipate energy. In fact, our adhesion energies are comparable to the values reported in an SFA study of MFP-5 (up to 14 mJ/m^2^),^40^ the most adhesive mussel foot protein.^2^ Collectively, these results demonstrate that films of Tren(Lys-1,2-HOPO)_3_ mediate robust, pH-tolerant adhesion between mica surfaces under water.

**Figure 3:**
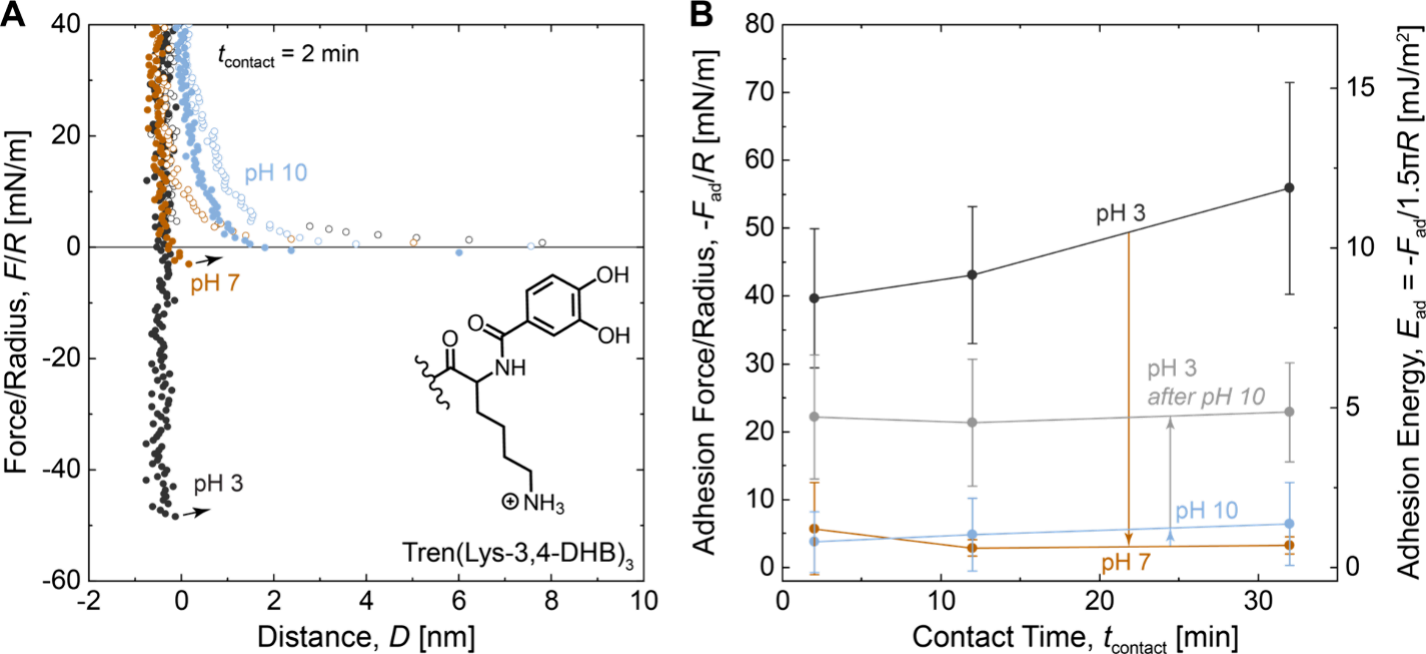
Effect of the pH on the adhesion of Tren(Lys-3,4-DHB)_3_. (A) Plots of force/radius *F*/*R* vs mica–mica separation distance *D*. Open circles correspond to approach; closed circles correspond to separation. (B) Plots of adhesion force/radius −*F*_ad_/*R* and adhesion energy *E*_ad_ vs contact time *t*_contact_. Measurements were performed in solutions of 50 mM acetate (pH 3), 50 mM phosphate (pH 7), or 50 mM carbonate (pH 10), each with 150 mM KNO_3_.

We next investigated the adhesion of Tren(Lys-3,4-DHB)_3_, a surface primer bearing a 3,4-DHB group structurally similar to the catechol of 3,4-dihydroxyphenylalanine (DOPA), but more resistant to oxidation.^8^ To evaluate the adhesion of Tren(Lys-3,4-DHB)_3_, we deposited the surface primer on mica and measured the adhesion force in the SFA. Figure 3A shows plots of force/radius vs distance for Tren(Lys-3,4-DHB)_3_ at pH 3, 7, and 10; Figure 3B shows the average values of adhesion force and energy. The force–distance plots show short-range repulsive forces, nanometer film thicknesses, and abrupt jumps from adhesive contact similar to the behavior of Tren(Lys-1,2-HOPO)_3_. However, upon increasing the pH from 3 to 7, Tren(Lys-3,4-DHB)_3_ shows a much greater decrease in adhesion force than Tren(Lys-1,2-HOPO)_3_. Further increasing the pH from 7 to 10 did not affect the adhesion force. Returning the pH from 10 to 3 resulted in partial recovery of the adhesion force (Figure 3B, gray circles), but to a lesser extent than for Tren(Lys-1,2-HOPO)_3_. Data from the Tren(Lys-3,4-DHB)_3_ reversibility experiments alone are shown in Figure S8. These results, and particularly the substantial drop in adhesion of Tren(Lys-3,4-DHB)_3_ at pH 7, indicate that Tren(Lys-1,2-HOPO)_3_ might be preferred over Tren(Lys-3,4-DHB)_3_ as an adhesive functionality for use in neutral solutions, including in physiological environments.

**Figure 4:**
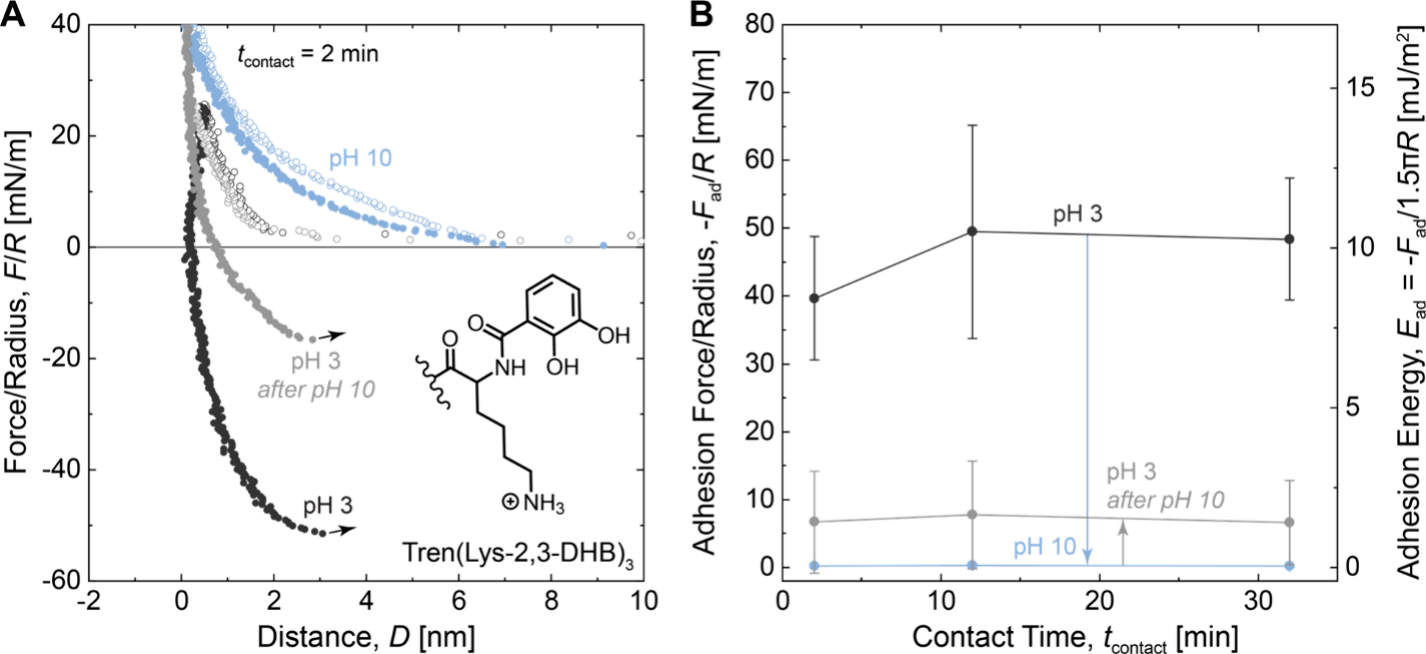
Reversibility of the adhesion after changes in the pH for Tren(Lys-2,3-DHB)_3_. (A) Plots of force/radius *F*/*R* vs mica–mica separation distance *D*. Open circles correspond to approach; closed circles correspond to separation. (B) Plots of adhesion force/radius -*F*_ad_/*R* and adhesion energy *E*_ad_ vs contact time *t*_contact_. Measurements were performed in solutions of 50 mM acetate (pH 3) or 50 mM carbonate (pH 10), each with 150 mM KNO_3_.

We also investigated the adhesion of Tren(Lys-2,3-DHB)_3_, a regioisomer of Tren(Lys-3,4-DHB)_3_, to test the influence of the catechol hydroxyl group position on adhesion. This work extends a previous study of Tren(Lys-2,3-DHB)_3_ that found decreasing adhesion with increasing pH up to pH 7,^32^ but did not investigate alkaline pH values or the reversibility of the changes in adhesion. We measured the adhesion of Tren(Lys-2,3-DHB)_3_ while changing the pH from 3 to 10, and then back to 3 (Figure 4). At pH 3, Tren(Lys-2,3-DHB)_3_ showed similar force–distance profiles and adhesion force to Tren(Lys-3,4-DHB)_3_ and Tren(Lys-1,2-HOPO)_3_. Increasing the pH to 10 eliminated adhesion. Upon returning the pH from 10 to 3, the adhesion of Tren(Lys-2,3-DHB)_3_ recovered slightly, but to a lesser extent than either Tren(Lys-3,4-DHB)_3_ or Tren(Lys-1,2-HOPO)_3_. We note that the apparent range of attractive interaction over several nm at each pH value in Figure 4 may result from misalignment of the optical path in the SFA, or cohesive interactions due to overadsorption of surface primers, as previous measurement of the same molecules^32^ showed abrupt jumps from contact similar to the data shown in Figures 2 and 3. We also note that we observed an increased range of repulsion of Tren(Lys-2,3-DHB)_3_ at pH 10 (Figure S9), but the experimental error associated with our deposition method precluded a detailed evaluation.

**Figure 5:**
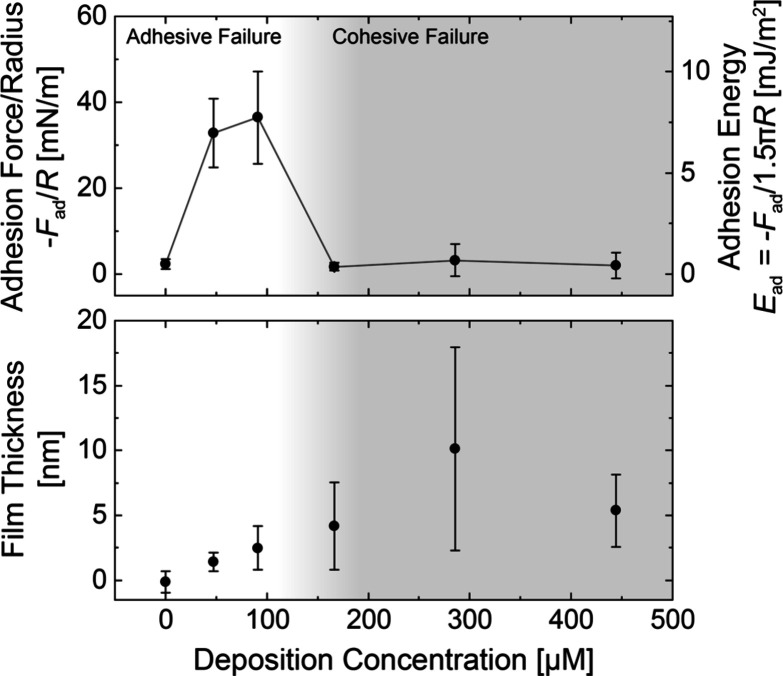
Properties of films of Tren(Lys-1,2-HOPO)_3_ vs deposition concentration. (Top) Adhesion force/radius −*F*_ad_/*R* and adhesion energy *E*_ad_ (*t*_contact_ = 2 min). (Bottom) Film thickness measured at 20 mN/m. Shading indicates the proposed transition between the adhesive and cohesive failure.

To interpret our data, we sought to identify the failure mode. During an adhesion measurement, failure can occur at the interface between the adhesive material and the substrate, known as adhesive failure, or within the bulk of the adhesive material, known as cohesive failure. It has been previously shown that the failure mode of surface primers in an SFA can be inferred from the dependence of adhesion force on deposition concentration.^34^ We therefore investigated films of Tren(Lys-1,2-HOPO)_3_ deposited at different concentrations. Figure 5 plots the average adhesion force (top) and film thickness at 20 mN/m compression (bottom) as functions of the deposition concentration of Tren(Lys-1,2-HOPO)_3_. Bare mica surfaces in buffer, without surface primers, showed weak adhesion (2 ± 1 mN/m) and a low film thickness (−0.1 ± 0.8 nm) (Figure S10). As the concentration of surface primers increased, the adhesion force first increased and then decreased, while the film thickness increased. At each concentration, the adhesion force was independent of the contact time within experimental error (Figure S11). Based on the abrupt decrease in adhesion force with increasing deposition concentration and the lack of dependence of adhesion force on contact time, we hypothesize that our results show a transition from adhesive failure to cohesive failure, indicated by the shaded region in Figure 5, implying that cohesion between films of Tren(Lys-1,2-HOPO)_3_ is weaker than adhesion of the films to mica. We propose that adhesion is maximized when incomplete films of primers on each surface interdigitate, allowing individual surface primers to bind to both mica surfaces, known as bridging interactions. We note that cohesive interactions between primers likely also contribute to the measured adhesion force. Due to the similarity in size, charge, and adhesion force of the surface primers tested, we expect Tren(Lys-3,4-DHB)_3_ and Tren(Lys-2,3-DHB)_3_ to show a similar dependence of failure mode on deposition concentration as seen for Tren(Lys-1,2-HOPO)_3_, with adhesive failure occurring for primers deposited at 50–100 μM. Because the data shown in Figures 2–4 correspond to surface primers deposited at 90 μM, we attribute the measured forces to adhesive failure.

Identifying the failure mode enables the measured adhesion to be attributed to intermolecular interactions with the surface. In our experiments, catechol analogs and the cationic amines of lysine and the Tren core are expected to bind to the mica surfaces and contribute to the adhesion force. We first estimated the relative contributions of these groups to adhesion at pH 3. Adhesion in acidic conditions has been reported for surface primers functionalized with lysyl amines and noncatechol aromatic groups (10 mN/m),^32^ and for the unfunctionalized Tren core with three primary amines (5 mN/m).^33^ Based on these measurements, we estimate that cationic amines account for approximately 5–10 mN/m of the adhesion in our measurements at pH 3, likely due to electrostatic interactions with the mica surface, which exhibits a negative zeta potential over the pH range tested here.^41^ We attribute the majority of the adhesion (≥30 mN/m) to the catechol analogs, consistent with a previous study showing that Tren(Lys-Lys-2,3-DHB)_3_ adhered less strongly than Tren(Lys-2,3-DHB)_3_, despite having twice as many lysine residues.^33^ We note that the estimated adhesive contribution of the catechol analogue is unexpected for Tren(Lys-1,2-HOPO)_3_ based on the written structure of 1,2-HOPO, which contains a single hydroxyl group. A similar surface primer functionalized with lysine and hydroxybenzamide, a functionality that contains one hydroxyl group, was found to adhere relatively weakly (10 mN/m).^32^ However, resonance of HOPO functionalities^42^ may enable additional hydrogen bonding, or possibly coordinate covalent bonding with aluminum atoms on the mica lattice.

We next considered the origin of the reduction in adhesion with increasing pH. As the pH increases, adhesion to mica might decrease by either deprotonation or oxidation of catechol analogs, both of which prevent hydrogen bond donation, with a possible additional contribution from deprotonation of cationic amines. However, deprotonation would be reversed upon returning to acidic conditions, whereas catechol oxidation is expected to be irreversible.^43^ In the case of Tren(Lys-1,2-HOPO)_3_, the changes in adhesion with pH were largely reversible (Figure 2 and Figure S7), suggesting that deprotonation, rather than oxidation, accounts for the decreased adhesion at pH 10. Although the p*K*_a_ values of ionizable groups of Tren(Lys-1,2-HOPO)_3_ have not been reported, the p*K*_a_ values of Tren(1,2-HOPO)_3_ (Table S6) suggest that the hydroxyl group of 1,2-HOPO may be protonated at pH 3 and deprotonated at pH 10. Also, HOPO oxidation is not expected at pH 10 based on prior cyclic voltammetry measurements.^44^ Unlike the reversible adhesion of Tren(Lys-1,2-HOPO)_3_, the adhesion of Tren(Lys-3,4-DHB)_3_ and Tren(Lys-2,3-DHB)_3_ only partially recovered upon changing from pH 10 to 3. The recovery of adhesion may be attributed to the protonation of catechol hydroxyl groups. At pH 10, one hydroxyl group on each DHB functionality of Tren(Lys-2,3-DHB)_3_ is expected to be deprotonated (Tables S6 and S7). Although p*K*_a_ values have not been reported for Tren(Lys-3,4-DHB)_3_, 3,4-dihydroxybenzoic acid (3,4-DHBA) has lower hydroxyl p*K*_a_ values than 2,3-DHBA (Table S6), suggesting that Tren(Lys-3,4-DHB)_3_ may be at least as deprotonated as Tren(Lys-2,3-DHB)_3_ at pH 10.

The irreversible decreases in adhesion of Tren(Lys-3,4-DHB)_3_ and Tren(Lys-2,3-DHB)_3_ may be attributed to oxidation of the catechol analogs in each compound. Interestingly, Tren(Lys-2,3-DHB)_3_ recovered less adhesion upon returning from pH 10 to 3 than Tren(Lys-3,4-DHB)_3_, suggesting that Tren(Lys-2,3-DHB)_3_ may oxidize more easily than Tren(Lys-3,4-DHB)_3_. To further investigate this observation, we prepared stock solutions of Tren(Lys-3,4-DHB)_3_ and Tren(Lys-2,3-DHB)_3_ at pH 10 and then adjusted the pH to 3 before depositing the primers on mica and measuring the adhesion (Figure S12). We found that Tren(Lys-2,3-DHB)_3_ prepared in this way showed no adhesion at pH 3, whereas Tren(Lys-3,4-DHB)_3_ showed adhesion similar to the values shown in Figure 3. Furthermore, the solution of Tren(Lys-2,3-DHB)_3_ at pH 10 showed a distinct color change characteristic of catechol oxidation and cross-linking,^26^ whereas Tren(Lys-3,4-DHB)_3_ was only mildly discolored. Collectively, our results indicate that Tren(Lys-3,4-DHB)_3_ is less susceptible to oxidation than Tren(Lys-2,3-DHB)_3_. This result is surprising, given that 3,4-DHBA oxidizes more readily than 2,3-DHBA in bulk solution,^8^ and suggests that oxidation of catechol analogs in the surface primers may be influenced by the local molecular environment. Furthermore, both Tren(Lys-3,4-DHB)_3_ and Tren(Lys-2,3-DHB)_3_ oxidize more readily than does Tren(Lys-1,2-HOPO)_3_. The resilient adhesion of Tren(Lys-1,2-HOPO)_3_ over changes in pH as well as the adhesion in neutral pH suggests the HOPO functionality as a candidate for achieving interfacial adhesion in physiological and marine environments.

## Conclusions

We tested the adhesion to mica of surface primers functionalized with catechol analogs and a cationic amine (e.g., lysine) in different environmental conditions. We found that dihydroxybenzamide (DHB) and hydroxypyridinone (HOPO) functionalities adhered strongly to mica under acidic conditions. However, the HOPO functionality showed robust adhesion at pH 3 and 7 and recovered adhesion after temporary exposure to pH 10, whereas the DHB functionalities lost adhesion after exposure to pH 10 and showed signs of irreversible oxidation. The adhesion and oxidation-resistance of the HOPO functionality make it a particularly promising candidate for further study; research on different HOPO derivatives is ongoing in our laboratories. In the context of adhesive design, the choice of catechol analog will depend on the need for oxidation-resistant interfacial adhesion balanced against the need for oxidative cross-linking for cohesion. Ultimately, we anticipate that the control of catechol oxidation will enable the development of catechol-based materials with robust adhesion in diverse environments.

## Supporting Information Available

Additional experimental details, materials, and methods, including synthesis, NMR characterization of compounds, and force spectroscopy data (PDF)
