# Supplementary material for: pH-Tolerant Wet Adhesion of Catechol Analogs
Source: ACS Appl Mater Interfaces. 2024 Apr 15;16(17):22689–95. doi: 10.1021/acsami.4c01740 (PMC11071048; doi:10.1021/acsami.4c01740)
Supplement: Supplementary file 1 — am4c01740_si_001.pdf [file am4c01740_si_001.pdf]

Supporting Information

pH-tolerant wet adhesion of catechol analogs

George D. Degen,[1†] Syeda Tajin Ahmed,[2†] Parker R. Stow,[3†]
Alison Butler,[3] Roberto C. Andresen Eguiluz[2,4]*

[1]Department of Chemical Engineering, University of California, Santa Barbara, CA 93106, USA.
[2]Department of Materials Science and Engineering, University of California, Merced, CA 95344, USA.
[3]Department of Chemistry and Biochemistry, University of California, Santa Barbara, CA 93106, USA.
[4]Health Sciences Research Institute, University of California, Merced, CA 95344, USA.

[†]Contributed equally

*Corresponding author: randreseneguiluz@ucmerced.edu

S1. Synthesis methods for Tren(Lys-3,4-DHB)$_3$ and Tren(Lys-1,2-HOPO)$_3$
Scheme S1. Synthesis scheme for Tren(Lys-3,4-DHB)$_3$ and Tren(Lys-1,2-HOPO)$_3$
Methods S1. Synthesis methods for Tren(Lys-3,4-DHB)$_3$ and Tren(Lys-1,2-HOPO)$_3$
Figure S1. ^{1}H NMR of Tren(Lys-3,4-DHB)$_3$
Figure S2. ^{13}C NMR of Tren(Lys-3,4-DHB)$_3$
Figure S3. ^{1}H-^{13}C HMBC NMR of Tren(Lys-3,4-DHB)$_3$
Table S1. NMR data for Tren(Lys-3,4-DHB)$_3$
Figure S4. ^{1}H NMR of Tren(Lys-1,2-HOPO)$_3$
Figure S5. ^{13}C NMR of Tren(Lys-1,2-HOPO)$_3$
Figure S6. ^{1}H-^{13}C HMBC NMR of Tren(Lys-1,2-HOPO)$_3$
Table S2. NMR data for Tren(Lys-1,2-HOPO)$_3$
Table S3. Experimental counts for pH adhesion studies
Table S4. Experimental counts for reversibility studies
Table S5. Experimental counts for concentration sweep of Tren(Lys-1,2-HOPO)$_3$
Figure S7. Reversibility of the adhesion of Tren(Lys-1,2-HOPO)$_3$
Figure S8. Reversibility of the adhesion of Tren(Lys-3,4-DHB)$_3$
Figure S9. Range of repulsion of the surface primers
Figure S10. Adhesion of mica surfaces in buffer
Figure S11. Adhesion and film thickness of Tren(Lys-1,2-HOPO)$_3$ vs deposition concentration
Table S6. pKa values of selected compounds and functional groups.
Table S7. Average deprotonation fraction and total charge of Tren(Lys-2,3-DHB)$_3$ at pH 3, 7, and 10.
Figure S12. Adhesion measurements of Tren(Lys-2,3-DHB)$_3$ and Tren(Lys-3,4-DHB)$_3$ deposited after incubation at pH 10.

S1. Synthetic methods for Tren(Lys-3,4-DHB)$_3$ and Tren(Lys-1,2-HOPO)$_3$

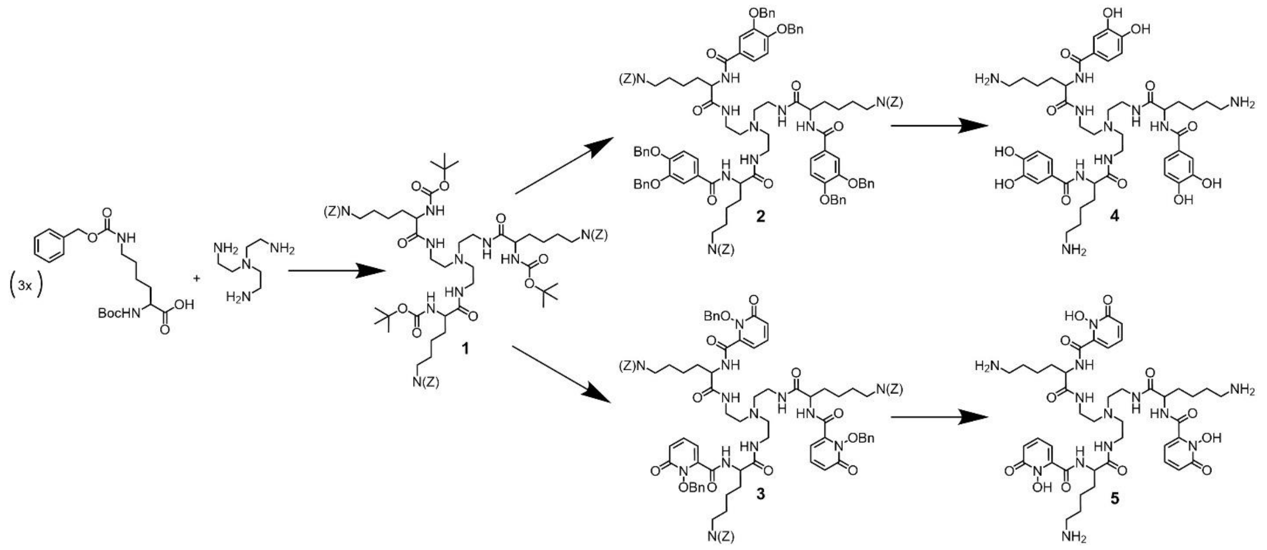

Scheme S1. Synthetic scheme for Tren(Lys-3,4-DHB)$_3$ (4) and Tren(Lys-1,2-HOPO)$_3$ (5)

General experimental procedures

^{13}C NMR spectroscopy was performed on a Bruker Advanced Neo 500 MHz spectrometer equipped with a prodigy BBO cryoprobe at RT. All ^{1}H, COSY, HMBC, HSQC NMR spectroscopy was performed on a Varian Unity 600 MHz spectrometer at RT. Chemical shifts were referenced through residual solvent peaks [^{1}H (DMSO-d_6) 2.50 ppm, ^{13}C (DMSO-d_6) 39.51 ppm]. Mass spectrometry analysis of synthetic products was carried out on a Waters/Micromass LCT Premier (UC Irvine) with isocratic MeOH flow at 0.2 mL/min and 10 μL injection volume.

General synthetic procedures

All reactions performed under an argon atmosphere were carried out using a high-vacuum line, standard Schlenk techniques, and dry solvents. DMF, DCM, and DMSO-d_6 were stored over 3 Å molecular sieves for at least 72 h prior to use. *N*,*N′*-diisopropylethylamine (DIPEA) was purified by distillation over ninhydrin (x3) and was subsequently stored over 3 Å molecular sieves. N_α-Boc-N_ε-Cbz-L-lysine was acquired from Bachem. All other reagents were purchased from Sigma-Aldrich. Tren-LysCAM,[1] 2,3-dibenzyloxybenzoic acid (Bn-2,3-DHBA),[2] and Bn-1,2-HOPO[3] were synthesized according to literature procedures. An analogous procedure for the synthesis of Bn-3,4-DHBA was carried out by substituting 3,4-DHBA for 2,3-DHBA.

Synthesis of N,N',N''-tris[N_{α}-Boc-N_{ε}-Cbz-L-lysinyl]tris(2-aminoethyl)amine, 1.

N_{α}-Boc-N_{ε}-Cbz-L-lysine (761 mg, 2.0 mmol) and N-hydroxysuccinimide (276 mg, 2.4 mmol) were dissolved in 15 mL of dry DMF under an argon atmosphere and cooled in an ice bath. Dicyclohexylcarbodiimide (495 mg, 2.4 mmol) was added at 0 ℃ and the flask was subsequently taken out of the ice bath and stirred at RT for four hours. Tris(2-aminoethyl)amine (74.9 µL, 0.5 mmol) and DIPEA (1044 µL, 6 mmol) were then added to the flask and the reaction was stirred overnight at RT. The following day the DCU byproduct was filtered off and the solvent was removed *in vacuo*. The crude reaction mixture was brought up in DCM and rinsed with sat. $NaHCO_3$ (30 mL, x3) and brine (30 mL). The organic layer was concentrated and then loaded onto a silica column. Purification by flash chromatography using a gradient of 2 – 4 % MeOH in DCM afforded **1** as a colorless solid. (76% yield). ^{1}H NMR (DMSO-d_6, 25 °C):): δ = 1.25 (m, 6H; CH_2), 1.34 (m,6H; CH_2), 1.36 (s, 27H; CH_3), 2.46 (s, 6H; CH_2) 2.95 (m, 6H; CH_2), 3.08 (m, 6H; CH_2), 3.84 (td, J = 8.3, 4.7, 6H; CH_2), 4.99 (s, 6H; CH_2), 6.76 (d, J = 8.1, 3H; NH), 7.20 (t, J = 5.5, 3H; NH), 7.28 – 7.37 (m, 15H; Ar-H), 7.67 (t, J = 5.5, 3H; NH) ppm. ^{13}C NMR (DMSO-d_6, 25 °C): δ = 22.8, 28.2, 29.1, 31.6, 36.9, 40.1, 53.2, 54.3, 65.1, 78.0, 127.7, 128.3, 137.3, 155.3, 156.1, 172.1 ppm. HRMS (ESI) *m/z* calculated for $C_{63}H_{97}N_{10}O_{15}$ + H^+: 1233.7135 [M+H^+]; found: 1233.7115.

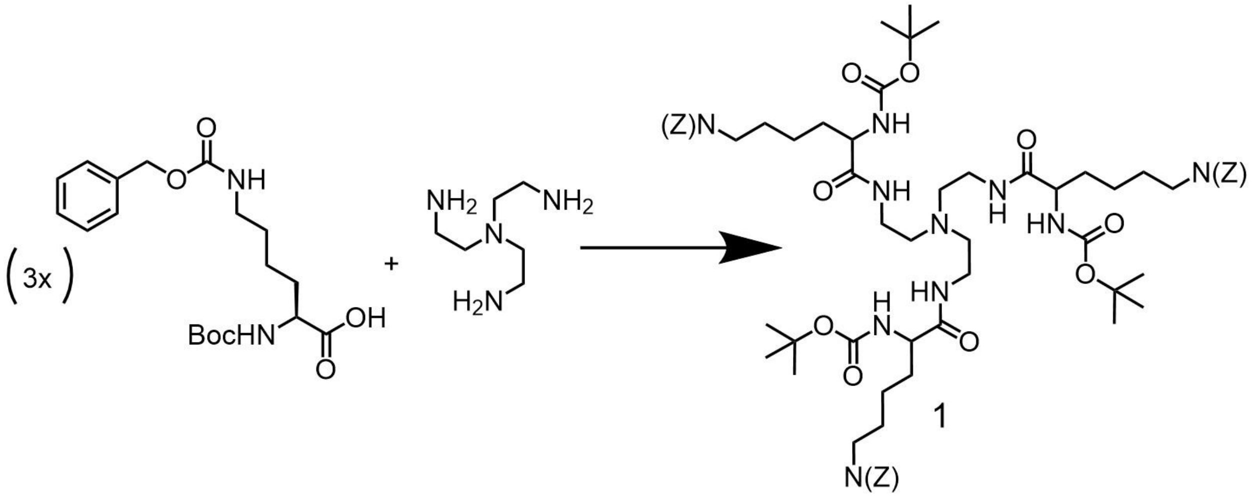

*Synthesis of N,N',N''-tris[N$_\alpha$-2,3-di(benzyloxy)benzoyl-N$_\varepsilon$-Cbz-L-lysinyl]tris(2-aminoethyl)amine (BnTren(Lys-3,4-DHB)$_3$), 2***.**

Compound **1** (370 mg, 0.3 mmol) was added to a dry flask under argon and dissolved in 6 mL dry DCM. The flask was cooled in an ice bath and 4 mL of TFA was added. After stirring for 1.5 h at RT, full deprotection of the boc groups was observed by TLC. Volatiles were removed *in vacuo* and the pale yellow oil was brought up in 5 mL of dry DMF. In a separate flask, 3,4-dibenzyloxybenzoic acid (341 mg, 0.99 mmol), HATU (376 mg, 0.99 mmol), and DIPEA (627 µL, 3.6 mmol) were added to 5 mL of dry DMF under an argon atmosphere and stirred for 3 min at RT. The contents of the first flask were then transferred to the reaction mixture via syringe and the reaction was left to stir overnight at RT. The reaction mixture was concentrated, loaded onto a silica column, and then purified by flash chromatography using a gradient of 1 – 3 % MeOH in DCM. Fractions were combined and concentrated to yield **2** as a white solid. (81% yield over 2 steps). **2** was immediately deprotected to yield **4** according to the procedure below.

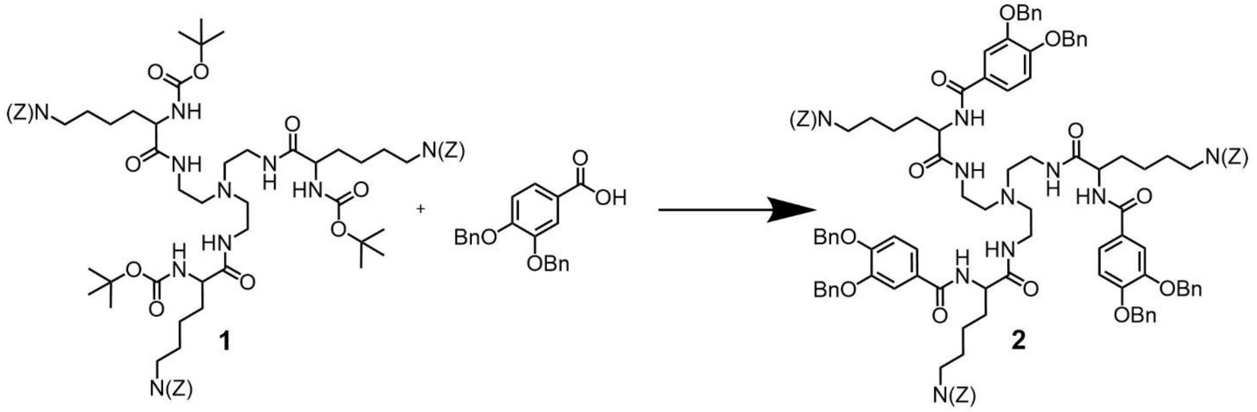

Synthesis of N,N',N''-tris[N$_{\alpha}$-1-(benzyloxy)-2-oxo-1,2-dihydropyridine]benzoyl-N$_{\varepsilon}$-Cbz-L-lysinyl]tris(2-aminoethyl)amine (BnTren(Lys-1,2-HOPO)$_3$), 3.

Compound **1** (370 mg, 0.3 mmol) was added to a dry flask under argon and dissolved in 6 mL dry DCM. The flask was cooled in an ice bath and 4 mL of TFA was added. After stirring for 1.5 h at RT, full deprotection of the boc groups was observed by TLC. Volatiles were removed *in vacuo* and the pale yellow oil was brought up in 5 mL of dry DMF. In a separate flask, 1,2-HOPOBn (245 mg, 0.99 mmol), HATU (376 mg, 0.99 mmol), and DIPEA (627 µL, 3.6 mmol) were added to 5 mL of dry DMF under an argon atmosphere and stirred for 3 min at RT. The contents of the first flask were then transferred to the reaction mixture via syringe and the reaction was left to stir overnight at RT. The reaction mixture was concentrated, loaded onto a silica column, and then purified by flash chromatography using a gradient of 4 – 10 % MeOH in DCM. Fractions were combined and concentrated to yield **3** as a white solid. (74% yield over 2 steps) ^{1}H NMR (DMSO-d_6, 25 °C): δ = 1.25 (m, 6H; CH_2), 1.33 (m, 6H; CH_2), 1.56 (m, 3H; CH_2), 1.61 (m, 3H; CH_2), 2.48 (m, 6H; CH_2), 2.87 (q, J = 6.9, 6H; CH_2), 3.12 (m, 6H; CH_2), 4.40 (td, J = 8.4, 5.4, 3H; CH), 4.98 (s, 6H; CH_2), 5.23 (dd, J = 32.4, 8.4, 6H; CH_2), 6.29 (dd, J = 6.8, 1.7, 3H; Ar-H), 6.64 (dd, J = 9.2, 1.7, 3H; Ar-H), 7.16 (t, J = 5.6, 3H; NH), 7.28 – 7.38 (m, 24H; ArH), 7.42 – 7.48 (m, 9H; Ar-H), 7.98 (t, J = 5.6, 3H; NH), 9.00 (d, J = 8.1, 3H; NH) ppm. **3** was immediately deprotected to yield **5** according to the procedure below.

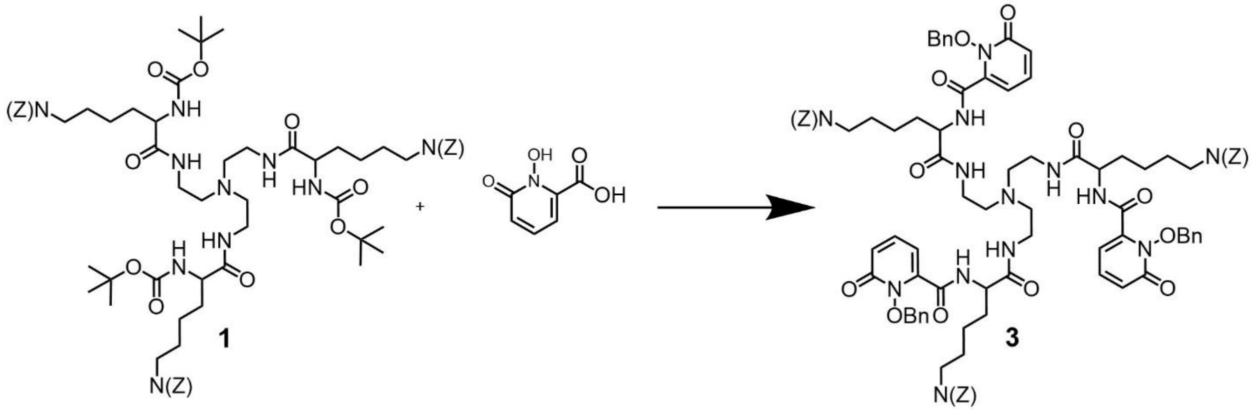

*N,N',N''-Tris[2,3-dihydroxybenzoyl-L-lysinyl]tris(2-aminoethyl)amine (Tren(Lys-3,4-DHB)$_3$), 4***.**
Compound **2** (376 mg, 0.2 mmol) was dissolved in 10 mL of 60% THF (aq.) + 0.5% acetic acid under an atmosphere of argon. 10% Pd/C (100 mg) was carefully added, and a balloon of hydrogen attached to a three-way flushing adapter was fitted to the round bottom. The atmosphere was evacuated and back-filled with hydrogen four times and stirred under an atmosphere of hydrogen for 24 h at RT. The catalyst was then filtered off, rinsed with 25 mL of DMF, and concentrated to yield a dark-red oil. The crude reaction, deemed mostly pure by NMR, was further purified by semi-preparative HPLC on a YMC-Actus 20 x 250 mm C18 ODS-AQ column using a linear gradient of 5% MeOH in ddH_2O (+0.1% trifluoroacetic acid) to 30% MeOH in ddH_2O (+0.1 % trifluoroacetic acid) over 25 min. HPLC fractions were concentrated and subsequently lyophilized to yield **4** as a white solid. (65% yield) ^{1}H NMR (DMSO-d_6, 25 °C): δ = 1.33 (m, 6H; CH_2), 1.54 (m, 6H; CH_2), 1.72 (m, 6H; CH_2), 2.75 (m, 6H; CH_2), 3.24 (s, 6H; CH_2), 3.43 (m, 6H; CH_2), 4.30 (td, *J* = 9.4, 4.9, 3H; CH), 6.77 (d, *J* = 8.3, 3H; Ar-H), 7.27 (dd, *J* = 8.3, 2.0, 3H; Ar-H), 7.34 (d, *J* = 2.1, 3H; Ar-H), 7.83 (s, 9H; NH_3), 8.19 (d, *J* = 7.5, 3H; NH), 8.27 (t, *J* = 5.4, 3H; NH) ppm. ^{13}C NMR (DMSO-d_6, 25 °C): δ = 22.8, 26.7, 30.7, 33.8, 38.7, 51.6, 53.4, 114.8, 115.5, 119.5, 125.0, 144.9, 148.7, 166.6, 173.0 ppm. HRMS (ESI) *m/z* calculated for $C_{45}H_{67}N_{10}O_{12}$ + H^+: 939.4940 [M+H^+]; found: 939.4960.

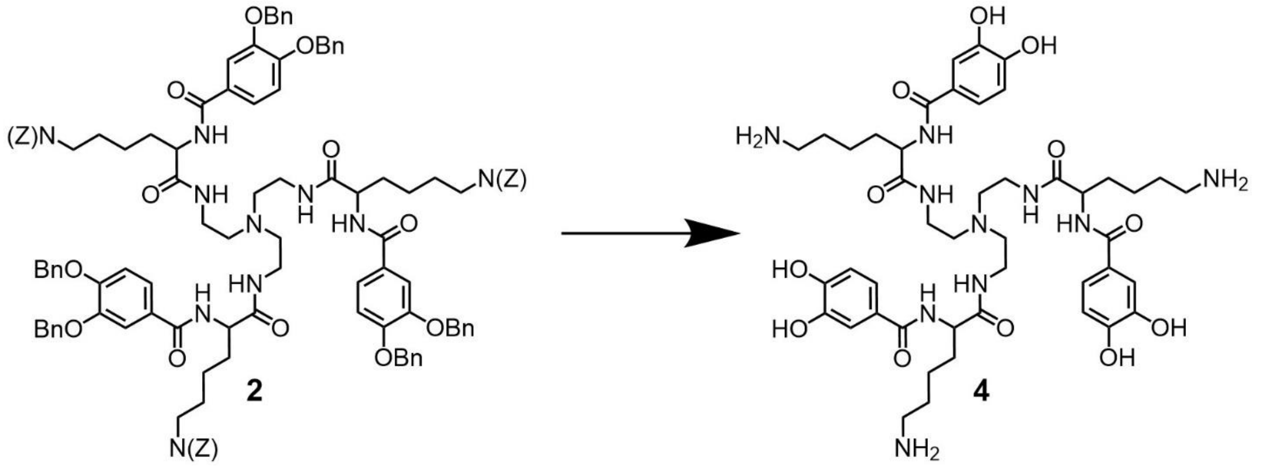

*N,N',N''-Tris[N_α-1-oxy-2-oxo-1,2-dihydropyridinebenzoyl-L-lysinyl]tris(2-aminoethyl)amine (Tren(Lys-1,2-HOPO)$_3$), 5***.**

Compound **3** (323 mg, 0.2 mmol) was dissolved in 5 mL of AcOH and 5 mL of conc. HCl. The reaction was stirred at RT for 72 hours and solvents were removed to yield a colorless oil. The crude reaction, deemed mostly pure by NMR, was further purified by semi-preparative HPLC on a YMC-Actus 20 x 250 mm C18 ODS-AQ column using a linear gradient of 5% MeOH in ddH_2O (+0.1% trifluoroacetic acid) to 30% MeOH in ddH_2O (+0.1 % trifluoroacetic acid) over 25 min. HPLC fractions were concentrated and subsequently lyophilized to yield **5** as a white solid. (65% yield). ^{1}H NMR (DMSO-d_6, 25 °C): δ = 1.36 (m, 6H; CH_2), 1.53 (m, 6H; CH_2), 1.60 (m, 6H; CH_2), 2.77 (m, 6H; CH_2), 3.17 (s, 6H; CH_2), 3.43 (s, 6H; CH_2), 4.33 (td, *J* = 8.8, 4.5, 3H; CH), 6.44 (dd, *J* = 6.9, 1.4, 3H; Ar-H), 6.62 (dd, *J* = 9.1, 1.4, 3H; Ar-H), 7.41 (dd, *J*= 6.9, 1.4, 3H; Ar-H), 7.85 (s, 9H; NH_3), 8.29 (t, *J* = 5.5, 3H; NH), 9.19 (d, *J* = 7.6, 3H; NH) ppm. ^{13}C NMR (DMSO-d_6, 25 °C): δ = 22.4, 26.6, 30.9, 33.9, 38.7, 51.6, 53.1, 104.9, 119.5, 136.9, 141.3, 157.5, 160.4, 171.7 ppm. HRMS (ESI) *m/z* calculated for $C_{42}H_{64}N_{13}O_{12}$ + H^+: 942.4797 [$M+H^+$]; found: 942.4785.

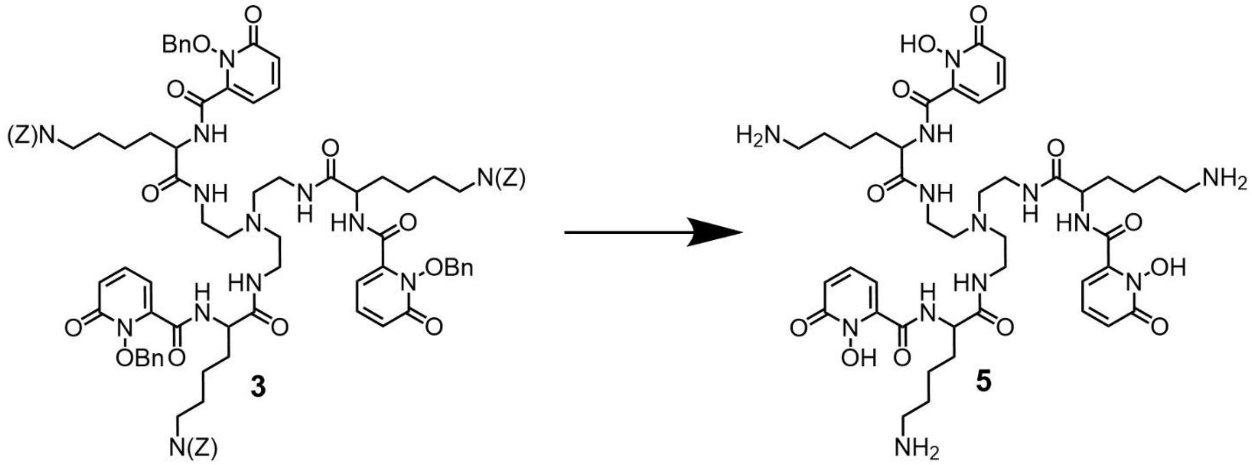

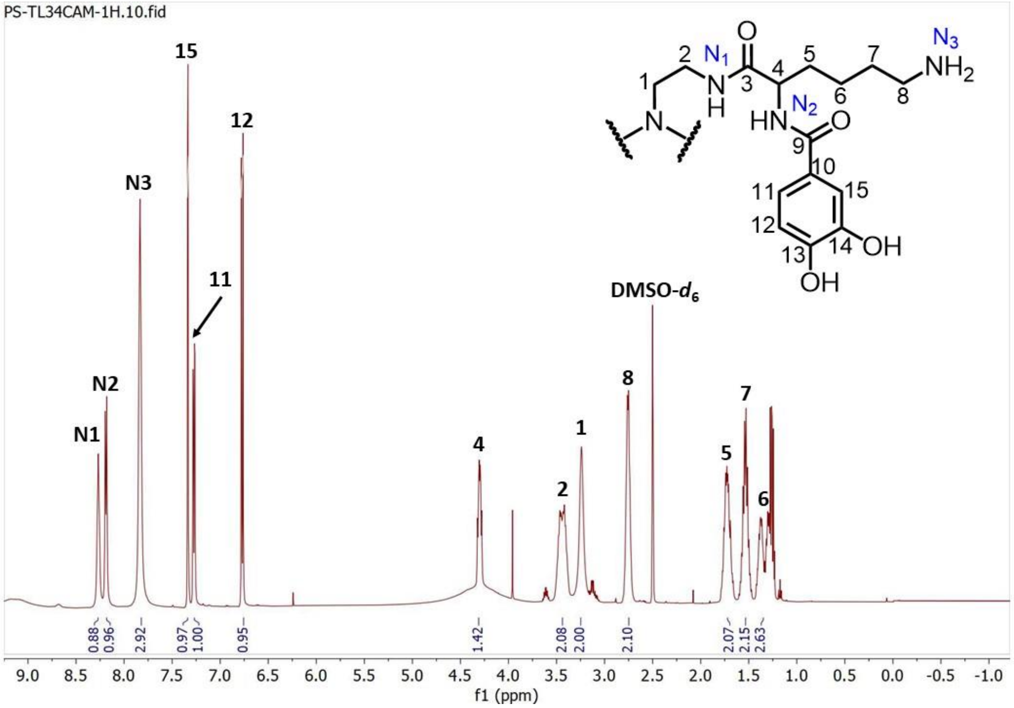

Figure S1. ^{1}H NMR of Tren(Lys-3,4-DHB)$_3$ in $(CD_3)_2SO$.

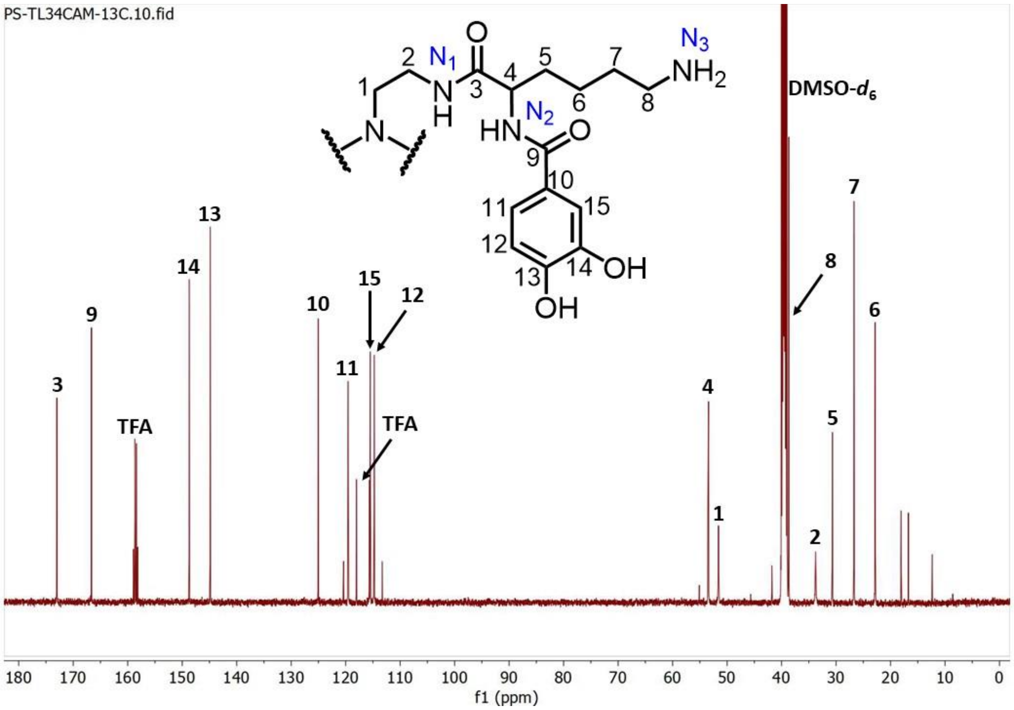

Figure S2. ^{13}C NMR of Tren(Lys-3,4-DHB)$_3$ in $(CD_3)_2SO$.

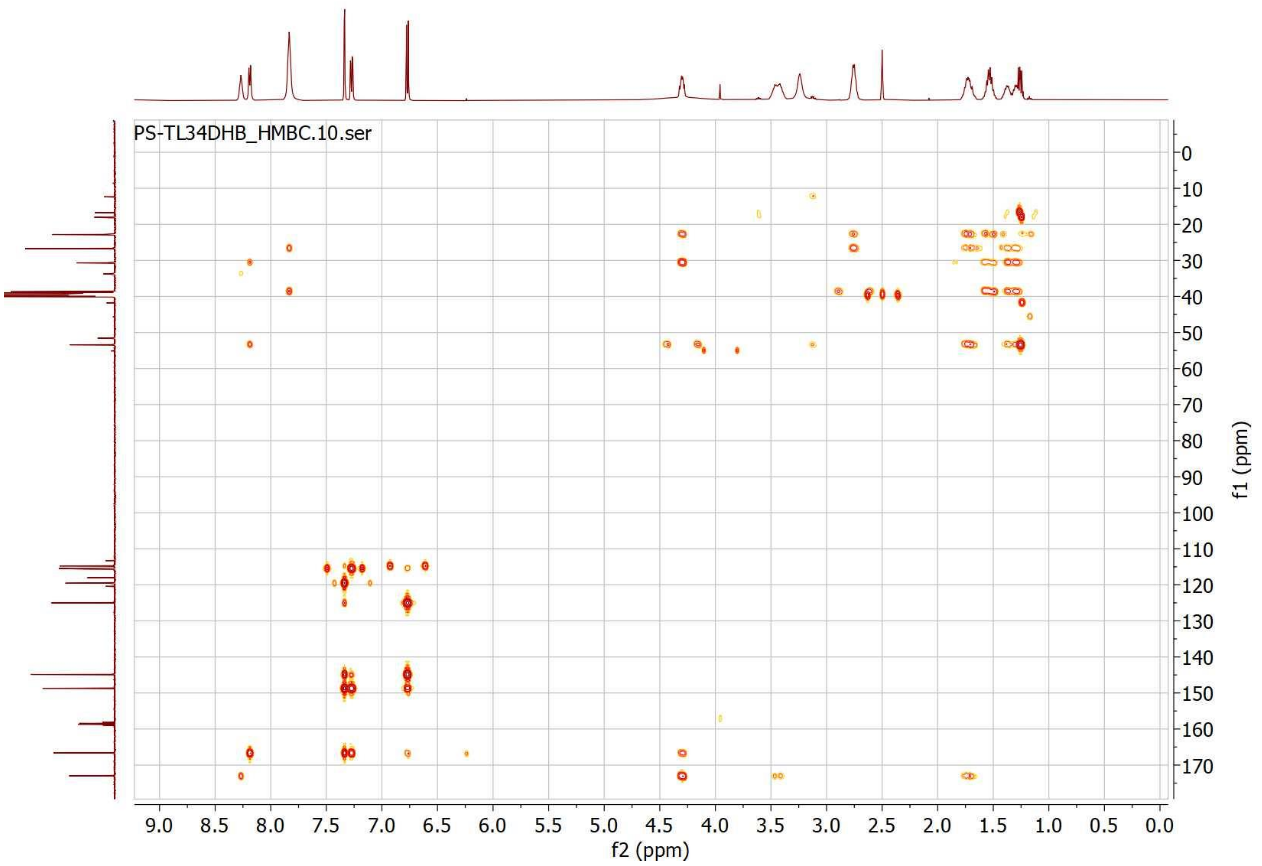

Figure S3. ^{1}H-^{13}C HMBC of Tren(Lys-3,4-DHB)$_3$ in $(CD_3)_2SO$.

Table S1. NMR data for Tren(Lys-3,4-DHB)$_3$ in $(CD_3)_2SO$. HMBC correlations are from proton(s) stated to the indicated carbon.

Tren(Lys-3,4-DHB)$_3$			
Position	δ_C, type	δ_H(J in Hz)	HMBC
1	51.6, CH_2	3.24, s	
2	33.8, CH_2	3.43, m	N1
3	173.0, C		2, 4, 5, N1
4	53.4, CH	4.30, td (9.4, 4.9)	5, 6, N2
5	30.7, CH_2	1.72, m	4, 6, 7, N2
6	22.8, CH_2	1.33, m	4, 5, 7, 8
7	26.7, CH_2	1.54, m	5, 6, 9, N3
8	38.7, CH_2	2.75, m	6, 7, N3
9	166.6, C		4, 11, 12, 15, N2
10	125.0, C		12, 15
11	119.5, CH	7.27, dd (8.3, 2.0)	15
12	114.7, CH	6.77, d (8.3)	15
13	148.7, CH		11, 12, 15
14	144.9, CH		11, 12, 15
15	115.5, CH	7.34, d (2.1)	11, 12
N1		8.27, t (5.4)	
N2		8.19, d (7.5)	
N3		7.83, s	

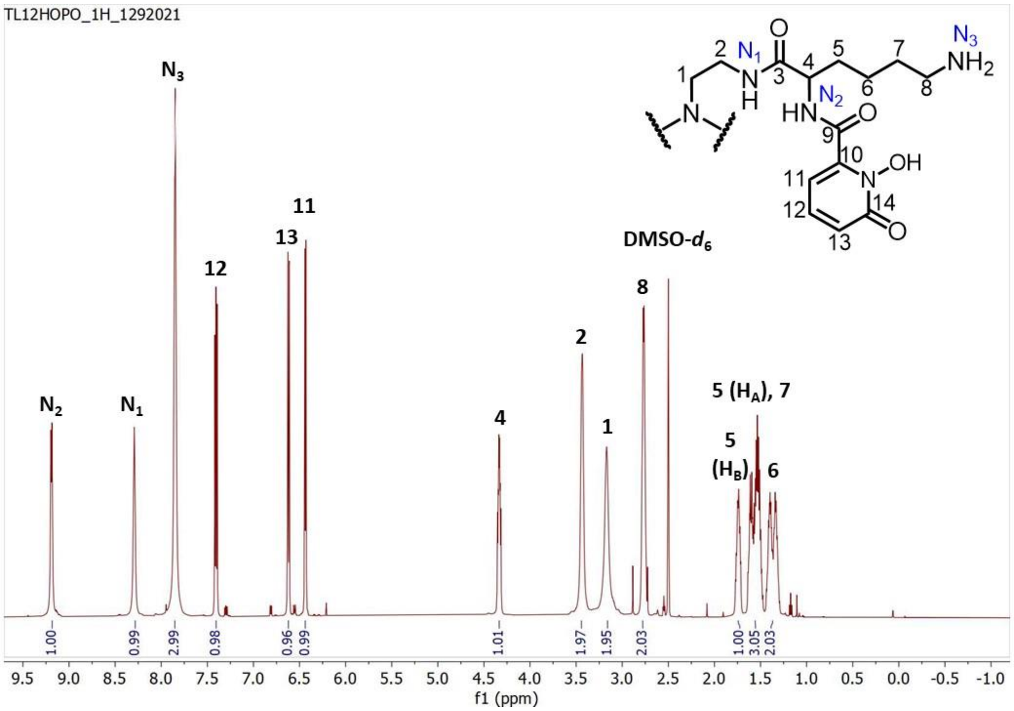

Figure S4. ^{1}H NMR of Tren(Lys-1,2-HOPO)$_3$ in $(CD_3)_2SO$.

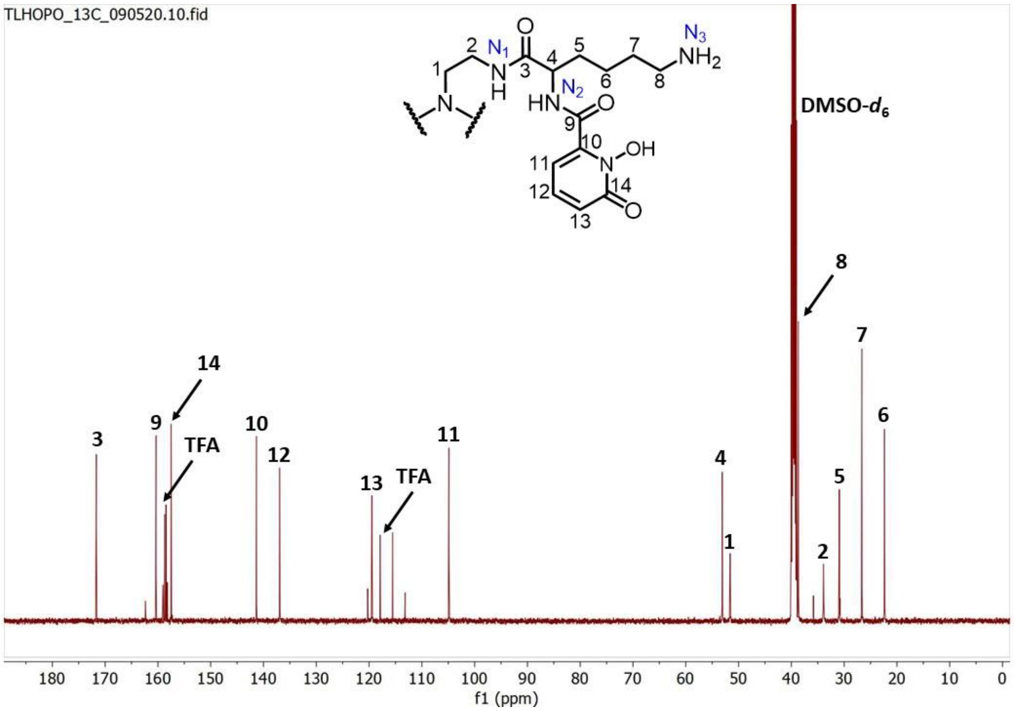

Figure S5. ^{13}C NMR of Tren(Lys-1,2-HOPO)$_3$ in $(CD_3)_2SO$.

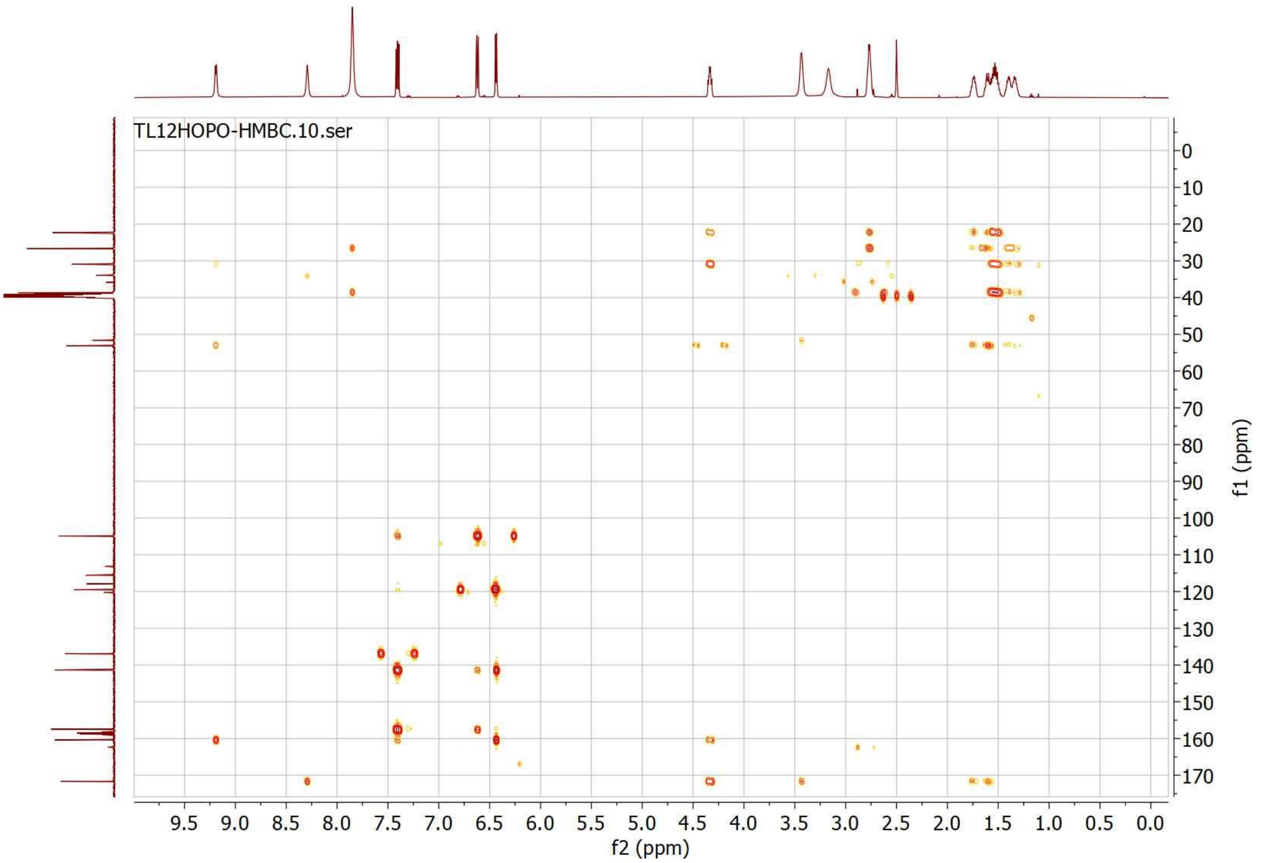

Figure S6. ^{1}H-^{13}C HMBC of Tren(Lys-1,2-HOPO)$_3$ in $(CD_3)_2SO$.

Table S2. NMR data for Tren(Lys-1,2-HOPO)$_3$ in $(CD_3)_2SO$. HMBC correlations are from proton(s) stated to the indicated carbon.

Tren(Lys-1,2-HOPO)$_3$			
Position	δ_C, type	δ_H(J in Hz)	HMBC
1	51.6, CH_2	3.17, s	2
2	33.9, CH_2	3.43, s	N1
3	171.7, C		2, 4, 5, N1
4	53.1, CH	4.33, td (8.8, 4.5)	5, 6, N2
5 (H_A)	30.9, CH_2	1.60, m	4, 6, 7, N2
5 (H_B)	30.9, CH_2	1.74, ddt (12.3, 10.8, 5.8)	4, 6, 7, N2
6	22.4, CH_2	1.36, m	4, 5, 7, 8
7	26.6, CH_2	1.53, m	5, 6, 8, N3
8	38.7, CH_2	2.77, m	6, 7, N3
9	160.4, C		4, 11, N2
10	141.3, C		11, 12, 13
11	104.9, CH	6.44, dd (6.9, 1.4)	12, 13
12	136.9, CH	7.41, dd (8.9, 6.9)	
13	119.3, CH	6.62, dd (9.1, 1.4)	11
14	157.6, C		12, 13
N1		8.29, t (5.5)	
N2		9.19, d (7.6)	
N3		7.85, s	

Table S3. Experimental counts for pH adhesion studies.

		$t_{contact}$ = 2 min	$t_{contact}$ = 12 min	$t_{contact}$ = 32 min
Tren(Lys-1,2-HOPO)$_3$	pH 3	12	7	6
	pH 7	4	4	4
	pH 10	8	6	4
Tren(Lys-3,4-DHB)$_3$	pH 3	12	9	6
	pH 7	6	3	3
	pH 10	7	5	5
Tren(Lys-2,3-DHB)$_3$	pH 3	11	7	6
	pH 10	6	5	4

Table S4. Experimental counts for reversibility studies.

		$t_{contact}$ = 2 min	$t_{contact}$ = 12 min	$t_{contact}$ = 32 min
Tren(Lys-1,2-HOPO)$_3$	pH 3	3	3	2
	pH 10	3	3	2
	pH 3	3	2	2
Tren(Lys-3,4-DHB)$_3$	pH 3	3	3	3
	pH 10	3	3	3
	pH 3	2	2	2
Tren(Lys-2,3-DHB)$_3$	pH 3	4	4	3
	pH 10	4	4	3
	pH 3	3	3	2

Table S5. Experimental counts for concentration sweep of Tren(Lys-1,2-HOPO)$_3$

Concentration (μM)	0	48	91	167	286	444
Number	13	3	4	3	4	4

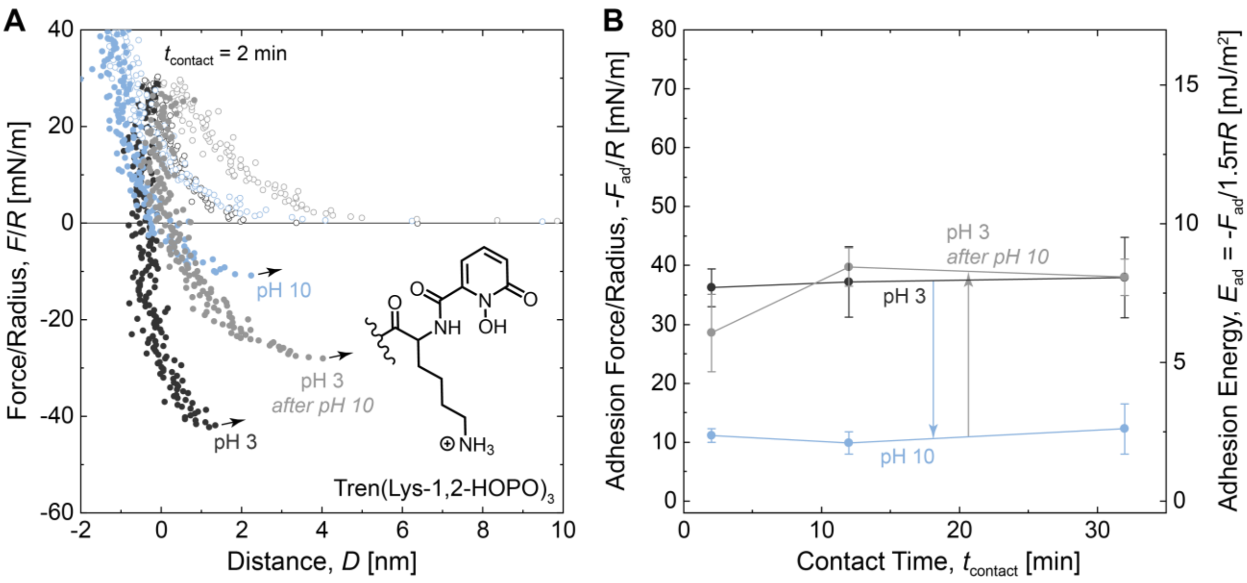

Figure S7. Reversibility of the adhesion of Tren(Lys-1,2-HOPO)$_3$. (A) Plots of force/radius F/R vs mica–mica separation distance D. Open circles correspond to approach; closed circles correspond to separation. (B) Plots of adhesion force/radius $-F_{ad}/R$ and adhesion energy E_{ad} vs contact time $t_{contact}$.

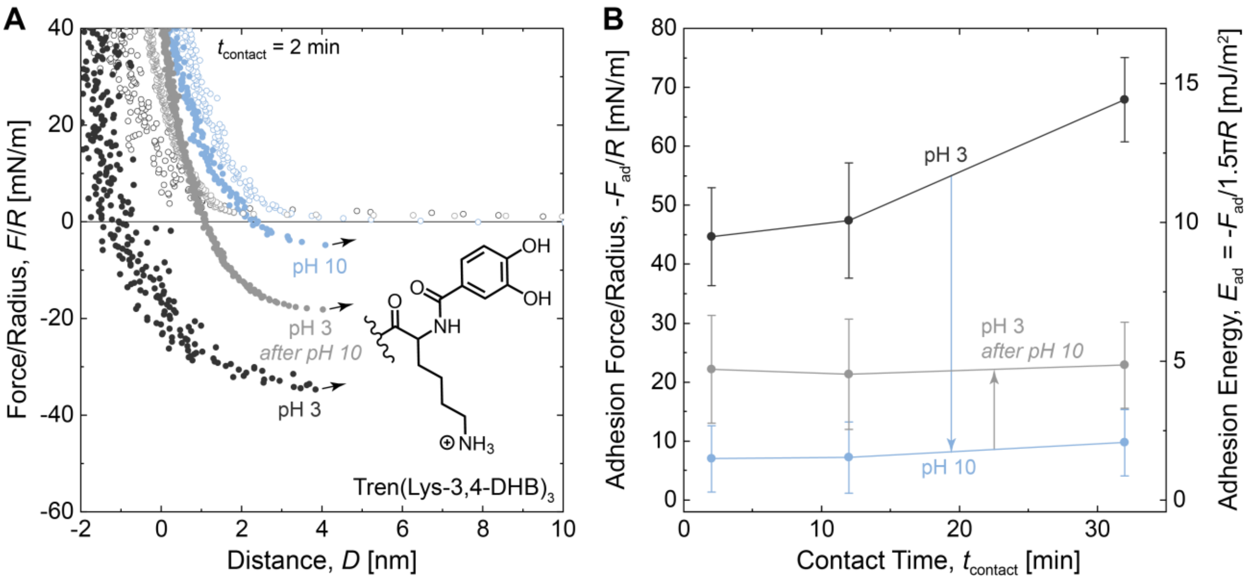

Figure S8. Reversibility of the adhesion of Tren(Lys-3,4-DHB)$_3$. (A) Plots of force/radius F/R vs mica–mica separation distance D. (B) Plots of adhesion force/radius $-F_{ad}/R$ and adhesion energy E_{ad} vs contact time $t_{contact}$. Open circles correspond to approach; closed circles correspond to separation.

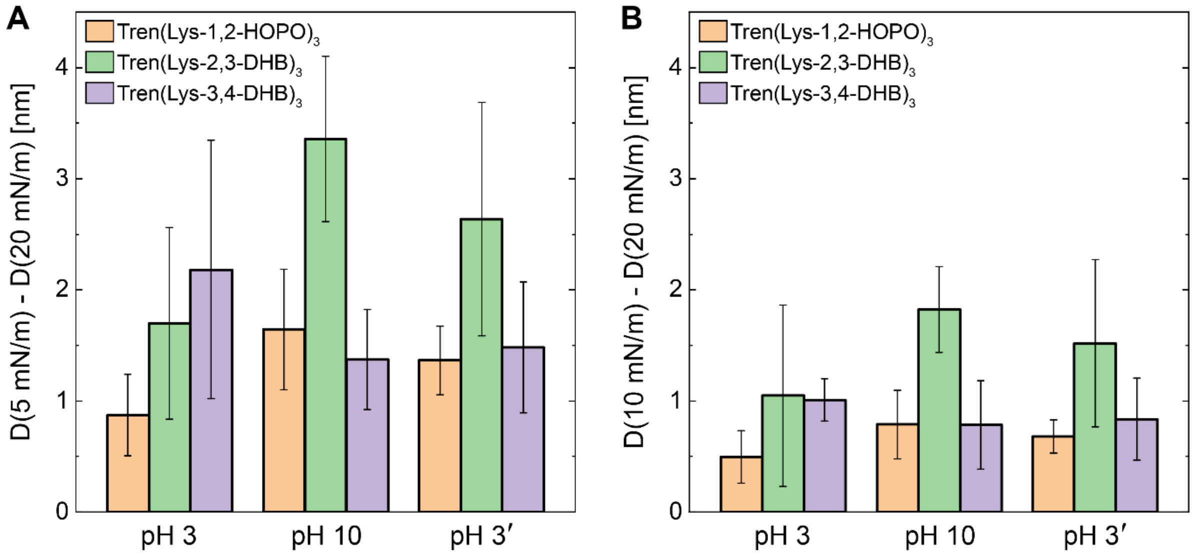

Figure S9. Range of repulsion of the surface primers, quantified by the difference in film thickness at different extents of compression.

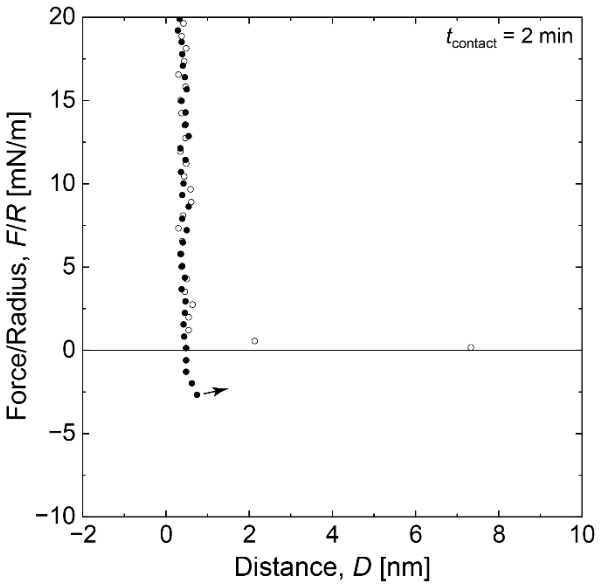

Figure S10. Adhesion of mica surfaces in buffer (50 mM acetate, 150 mM KNO3, pH 3). Open circles correspond to approach; closed circles correspond to separation.

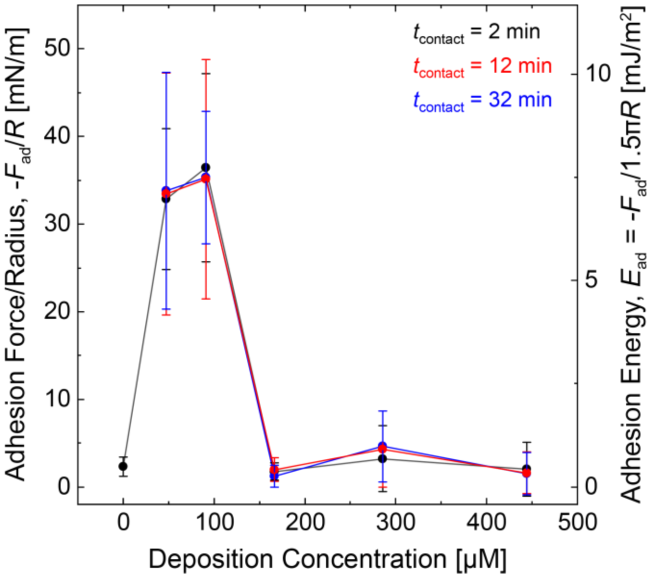

Figure S11. Adhesion of Tren(Lys-1,2-HOPO)3 vs deposition concentration for different contact times.

Table S3. pKa values of selected compounds and functional groups.

	Hydroxyl 1	Hydroxyl 2	Lysyl Amine	Tertiary Amine
2,3-DHBA[4]	10.0	13.1	-	-
Chrysobactin[5]	6.7	12.1	10.6	-
Tren(2,3-DHB)$_3$[6]	6.71, 8.61, 8.75	11.3, 12.1, 12.9	-	5.88
Tren(Lys-2,3-DHB)$_3$[7]	6.24, 6.72, 7.37	11.3, 12.1, 12.9	>10	5.22
3,4-DHBA[4]	8.7	12.6	-	-
Tren(Lys-3,4-DHB)$_3$	Not Reported			
1,2-HOPO[8]	5.8	-	-	-
Tren(1,2-HOPO)$_3$[9,10]	3.60, 4.32, 5.62	-	-	7.16
Tren(Lys-1,2-HOPO)$_3$	Not Reported			

Table S4. Average deprotonation fractions of functional groups in Tren(Lys-2,3-DHB)$_3$ at pH 3, 7, and 10 calculated with the Henderson–Hasselbalch equation using the pKa values given in **Table S3**. The final column shows the total charge of Tren(Lys-2,3-DHB)$_3$, calculated by summing the average charges of the six hydroxyl groups, three lysyl amines, and one tertiary amine.

pH	Hydroxyl 1	Hydroxyl 2	Lysyl Amine	Tertiary Amine	Total Charge
3	N/A	N/A	N/A	0.01	3.99
7	0.60	N/A	N/A	0.98	1.22
10	1.00	0.02	0.20	1.00	-0.66

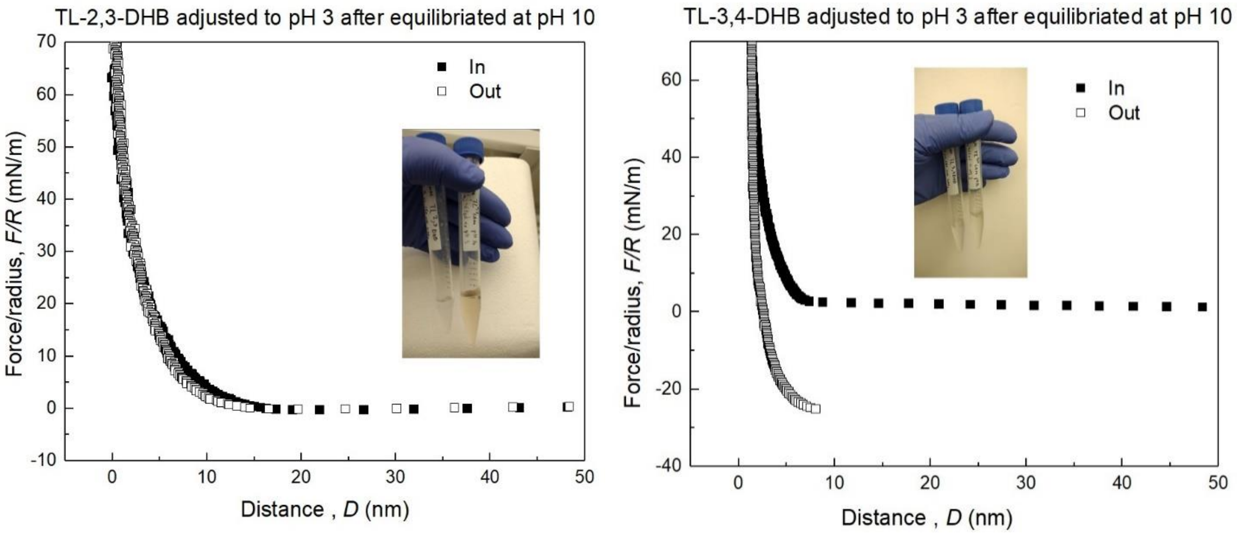

Figure S12. Force–distance measurements of Tren(Lys-2,3-DHB)$_3$ (left) and Tren(Lys-3,4-DHB)$_3$ (right). The surface primers were incubated at pH 10, and the solution was then adjusted to pH 3 prior to depositing the primers and measuring the adhesion force. The plots show that Tren(Lys-2,3-DHB)$_3$ adsorbs to mica at pH 3 after exposure to pH 10, but does not mediate adhesion. By contrast, Tren(Lys-3,4-DHB)$_3$ mediates an adhesion force similar to the force measured after cycling the pH from 3 to 10 to 3 (**Figure S8**). Insets show photos of the surface primers at pH 3 (left tube) and the primers after incubation at pH 10, followed by adjusting the pH 3 (right tube). Tren(Lys-2,3-DHB)$_3$ showed a clear color change after incubation at pH 10, consistent with oxidation of catecholic compounds.

References

(1) Maier, G. P.; Rapp, M. V; Waite, J. H.; Israelachvili, J. N.; Butler, A. Adaptive Synergy between Catechol and Lysine Promotes Wet Adhesion by Surface Salt Displacement. *Science* **2015**, *349* (6248), 625–628.

(2) Leydier, A.; Lin, Y.; Arrachart, G.; Turgis, R.; Lecerclé, D.; Favre-Reguillon, A.; Taran, F.; Lemaire, M.; Pellet-Rostaing, S. EDTA and DTPA Modified Ligands as Sequestering Agents for Uranyl Decorporation. *Tetrahedron* **2012**, *68* (4), 1163–1170.

(3) Daumann, L. J.; Tatum, D. S.; Snyder, B. E. R.; Ni, C.; Law, G. L.; Solomon, E. I.; Raymond, K. N. New Insights into Structure and Luminescence of EuIII and SmIII Complexes of the 3,4,3-LI(1,2-HOPO) Ligand. *J. Am. Chem. Soc.* **2015**, *137* (8), 2816–2819.

(4) Maier, G. P.; Bernt, C. M.; Butler, A. Catechol Oxidation: Considerations in the Design of Wet Adhesive Materials. *Biomater. Sci.* **2018**, *6* (2), 332–339.

(5) Tomišić, V.; Blanc, S.; Elhabiri, M.; Expert, D.; Albrecht-Gary, A. M. Iron(III) Uptake and Release by Chrysobactin, a Siderophore of the Phytophatogenic Bacterium Erwinia Chrysanthemi. *Inorg. Chem.* **2008**, *47* (20), 9419–9430.

(6) Rodgers, S. J.; Lee, C. W.; Ng, C. Y.; Raymond, K. N. Ferric Ion Sequestering Agents. 15. Synthesis, Solution Chemistry, and Electrochemistry of a New Cationic Analogue of Enterobactin. *Inorg. Chem.* **1987**, *26* (10), 1622–1625.

(7) Dertz, E. A.; Xu, J.; Raymond, K. N. Tren-Based Analogues of Bacillibactin: Structure and Stability. *Inorg. Chem.* **2006**, *45* (14), 5465–5478.

(8) Zhou, T.; Winkelmann, G.; Dai, Z. Y.; Hider, R. C. Design of Clinically Useful Macromolecular Iron Chelators. *J. Pharm. Pharmacol.* **2011**, *63* (7), 893–903.

(9) Jocher, C. J.; Moore, E. G.; Xu, J.; Avedano, S.; Botta, M.; Aime, S.; Raymond, K. N. 1,2-Hydroxypyridonates as Contrast Agents for Magnetic Resonance Imaging: TREN-1,2-HOPO. *Inorg. Chem.* **2007**, *46* (22), 9182–9191.

(10) Xu, J.; Churchill, D. G.; Botta, M.; Raymond, K. N. *Inorg. Chem*. **2004**, *43*, 5492−5494
